# On pandemics and the duty to care: whose duty? who cares?

**DOI:** 10.1186/1472-6939-7-5

**Published:** 2006-04-20

**Authors:** Carly Ruderman, C Shawn Tracy, Cécile M Bensimon, Mark Bernstein, Laura Hawryluck, Randi Zlotnik Shaul, Ross EG Upshur

**Affiliations:** 1 Primary Care Research Unit, Department of Family and Community Medicine, Sunnybrook Health Sciences Centre, 2075 Bayview Ave., Room E3-49, Toronto, ON M4N 3M5, Canada; 2 Joint Centre for Bioethics, University of Toronto, 88 College St., Toronto, ON M5G 1L4, Canada; 3 Division of Neurosurgery, Toronto Western Hospital, 399 Bathurst St., Toronto, ON M5T 2S8, Canada; 4 Interdepartmental Division of Critical Care, University Health Network, 200 Elizabeth St., Toronto, ON M5G 2C4, Canada; 5 Bioethics Department, The Hospital for Sick Children, 555 University Ave., Toronto, ON M5G 1X8, Canada; 6 Department of Family and Community Medicine, University of Toronto, 256 McCaul St., Toronto, ON M5T 2W5, Canada; 7 Department of Public Health Sciences, University of Toronto, 155 College St., Toronto, ON M5S 1A8, Canada

## Abstract

**Background:**

As a number of commentators have noted, SARS exposed the vulnerabilities of our health care systems and governance structures. Health care professionals (HCPs) and hospital systems that bore the brunt of the SARS outbreak continue to struggle with the aftermath of the crisis. Indeed, HCPs – both in clinical care and in public health – were severely tested by SARS. Unprecedented demands were placed on their skills and expertise, and their personal commitment to their profession was severely tried. Many were exposed to serious risk of morbidity and mortality, as evidenced by the World Health Organization figures showing that approximately 30% of reported cases were among HCPs, some of whom died from the infection. Despite this challenge, professional codes of ethics are silent on the issue of duty to care during communicable disease outbreaks, thus providing no guidance on what is expected of HCPs or how they ought to approach their duty to care in the face of risk.

**Discussion:**

In the aftermath of SARS and with the spectre of a pandemic avian influenza, it is imperative that we (re)consider the obligations of HCPs for patients with severe infectious diseases, particularly diseases that pose risks to those providing care. It is of pressing importance that organizations representing HCPs give clear indication of what standard of care is expected of their members in the event of a pandemic. In this paper, we address the issue of special obligations of HCPs during an infectious disease outbreak. We argue that there is a pressing need to clarify the rights and responsibilities of HCPs in the current context of pandemic flu preparedness, and that these rights and responsibilities ought to be codified in professional codes of ethics. Finally, we present a brief historical accounting of the treatment of the duty to care in professional health care codes of ethics.

**Summary:**

An honest and critical examination of the role of HCPs during communicable disease outbreaks is needed in order to provide guidelines regarding professional rights and responsibilities, as well as ethical duties and obligations. With this paper, we hope to open the social dialogue and advance the public debate on this increasingly urgent issue.

## Background

In 2003, the world witnessed the spread of a novel and deadly virus, namely SARS CoV. The health care workers (HCWs) and hospital systems that bore the brunt of the SARS outbreak continue to struggle with the aftermath of the crisis. Indeed, HCWs – both in clinical care and in public health – were severely tested by SARS. Unprecedented demands were placed on their skills and expertise, and their personal commitment to their profession was severely tried. Many were exposed to serious risk of morbidity and mortality; indeed, approximately 30% of reported cases were among HCWs, some of whom died from the infection [1].

As a number of commentators have noted, SARS exposed the vulnerabilities of our current health care systems and governance structures [2-4]. The aftermath of SARS and the spectre of pandemic avian influenza make imperative the need to consider the obligations of HCWs for patients with severe infectious diseases, particularly diseases that pose risks to those providing care. It is of pressing importance that organizations representing HCWs – professionals and non-professionals alike – give clear indication of what standard of care is expected of their members in the event of a pandemic.

Many experts believe that the SARS outbreak was merely a preview of the next flu pandemic that is soon to arrive, possibly from an avian influenza virus [5]. Quite clearly, avian flu threatens to be more widespread than SARS, with the potential to become a truly global pandemic. An honest and critical examination of the role of HCWs during such a crisis is needed in order to provide guidelines regarding professional rights and responsibilities, as well as ethical duties and obligations [6].

In this paper, we address the issue of special obligations of health care professionals (HCPs) during an infectious disease outbreak. We contend that there is a pressing need to clarify the rights and responsibilities of HCPs, especially in the current context of pandemic flu preparedness. Moreover, we argue that these rights and responsibilities would best be codified in professional codes of ethics. Finally, we present a brief historical account of the treatment of the duty to care in professional health care codes of ethics with the intention of opening the social dialogue and advancing the public debate on this increasingly relevant issue.

## Discussion

### Is there a problem?

Given that the response by HCPs to the SARS crisis was generally regarded as exemplary, one might ask whether an ethical problem truly exists. There is little doubt that the vast majority of HCPs performed their jobs admirably under considerable stress and significant personal risk. Many HCPs provided exemplary care, and still others behaved in truly heroic fashion. So why, then, formally problematize something that is not a problem?

As noted, many HCPs acted in a supererogatory manner during the SARS outbreak [7], none more so than Dr. Carlo Urbani of the World Health Organization, who himself died of SARS after being exposed to the yet unknown virus in the course of carrying out his professional duties. Likewise, scores of nurses, doctors, respiratory technicians, and other professional and non-professional health workers laboured extremely long hours at personal risk. This demonstration of going above and beyond the call of duty, which proved necessary to control the disease, was highly morally commendable.

At the same time, however, serious concerns did surface during SARS about the extent to which HCPs would tolerate risks of infection [8,9]. Some baulked at providing care to those infected with the unknown virus. In some circumstances, staffing became an issue in SARS wards and assessment centres; indeed, failure to report for duty during the outbreak resulted in the permanent dismissal of some hospital staff. As a consequence, the risk that was faced during SARS was not distributed equitably, and those HCPs who volunteered to provide care faced the greatest exposure.

Following the outbreak, many of those who treated SARS patients raised concerns about the protections that were provided to safeguard their own health and that of their family members. Conflicting obligations were another significant concern. HCPs are bound by an ethic of care, therefore, obligations to the patient's well-being should be primary. At the same time, however, HCPs have competing obligations to their families and friends, whom they feared infecting, in addition to obligations to themselves and to their own health (particularly those with special vulnerabilities, such as a co-morbid condition). During SARS, some HCPs questioned their choice of career; indeed, some decided to leave their profession and pursue new ventures, indicating an unwillingness or inability to care for patients in the face of risk. Recent survey data from the U.S. indicate that there exists mixed views on the duty to care for patients during infectious disease outbreaks [10].

What is clear is this: the issue of duty to care has emerged as a matter of paramount concern among health care professionals, hospital administrators, public policy makers, and bioethicists [11-14].

### Do health care professionals have special obligations during infectious disease outbreaks?

The ethical foundations of the duty to provide care are grounded in several longstanding ethical principles. Foremost among these is the principle of beneficience, which recognizes and defines the special moral obligation on the part of HCPs to further the welfare of patients and to advance patients' well-being. In modern health care, it is commonly understood and generally accepted that the principle of beneficence constitutes a foundational principle of the patient-provider relationship [15].

For the HCP in general, and for the physician in particular, there are a number of compelling reasons to provide care in the context of an infectious disease outbreak. Clark [12] has recently outlined three such reasons:

#### 1. The ability of physicians and health care professionals to provide care is greater than that of the public, thus increasing the obligation to provide care

Although self-care and self-protection, as well as the care and protection of friends and family members, are acknowledged in pandemic plans, it is evident that the expertise of HCPs is an integral and principal component of the response to a pandemic. There is no other sector of society that can be legitimately expected to fulfil this role and to assume this level of risk.

#### 2. By freely choosing a profession devoted to care of the ill, health care professionals have assumed risk

Arguably, HCPs have consented to greater than average risk by their very choice of profession. While it may be granted that the risk of contracting an infectious disease was likely not a concern for a generation of prospective health care workers, any informed reading of the medical literature in the last 20 years has shown that infectious diseases remain ubiquitous and problematic – notwithstanding overly-optimistic statements regarding the future threat of infectious diseases. It is therefore not unreasonable to argue that HCPs were aware of the greater than average risks posed by their choice of profession.

#### 3. The profession is legitimated by social contract and therefore its members should be available in times of emergency

In publicly-funded health care systems, such as those found in many Western societies, there is a strong claim for a social contract between the HCP and society. It is a reasonable and legitimate expectation by the public that HCPs will respond in an infectious disease emergency. Society has granted and permits professions to be self-regulating on the understanding that such a response would occur.

### The role of professional codes of ethics

One of the characteristics of a self-regulating profession is the development of standards of practice, sometimes referred to as best practice guidelines. These standards are articulated in professional codes of ethics, which are developed on the basis of the fundamental principles and values of the particular profession, as is the case, for instance, with respect to the codes of ethics that were developed long ago in medicine and nursing. Indeed, the code of ethics has a long and respected tradition in the health professions and today most, if not all, the various health and social care professions have codes of ethics in place to provide guidance to their members.

The code of ethics is sometimes referred to as an instrument of "soft law," owing to its non-legislative nature [16]. As such, in the health care professions, codes of ethics should be interpreted as guides for ethical reasoning and frameworks for the treatment of individual patients, rather than as substitutes for such reasoning or as an absolute mandate [17]. At the same time, a code that is too vague can render it ineffectual and irrelevant. In an era in which health care and technology are evolving at a rapid pace, efforts are necessary to ensure that codes of ethics remain current, practical, and concordant with public expectations.

An informative and comprehensible code of ethics has numerous tangible benefits. Perhaps the greatest benefit would be to dispel confusion and uncertainty for HCPs concerning their professional rights and responsibilities as regards the duty to care. Of course, a detailed treatment of the issue in professional codes of ethics would also serve to increase awareness and comfort levels, perhaps resulting in increased willingness to provide care in uncertain and risky conditions [18]. Additionally, codes guiding professional conduct may effectively serve as norms of standards recognizable and enforceable by law, acting as the foundation of legal obligations and decisions [16].

Finally, codes of ethics also serve as potent forms of symbolic communication to the public that is served by the professions. By making explicit the values that health care professions represent, professional codes of ethics can reassure the public that the trust invested in the professions is justified and legitimate, as is properly noted in the following excerpt from the College of Nurses of Ontario *Practice Standard on Ethics*:

"Nurses have a commitment to the nursing profession. Being a member of the profession brings with it the respect and trust of the public. To continue to deserve this respect, nurses have a duty to uphold the standards of the profession.... As members of a self-regulating profession, nurses also have a commitment to help regulate nursing to protect the public's right to quality nursing services. It is in the public's interest that the profession continue to regulate itself by developing and changing the methods of self-regulation to meet the changes in health care and society. Nurses have an obligation to participate in the effective evolution of self-regulation. Self-regulation is a privilege and each nurse is accountable for the responsibilities that accompany this privilege."[19]

### What do current codes of ethics say regarding duty to care during epidemics?

It is of no small concern that many current professional codes of ethics fail to provide explicit guidance sufficient to set policy or assure the public in the event of an infectious disease outbreak. The Canadian Medical Association, for instance, released a revised *Code of Ethics *in 2004, one year after the SARS pandemic in which Canada was particularly affected [20]. Despite the seemingly fortuitous timing of the publication, the revised *Code *is, quite astoundingly, altogether silent on physicians' duty to care, which might be described as the first among equals of the myriad ethical dilemmas that emerged during the global outbreak.

The key revision in the 2004 edition of the CMA *Code *was the addition of the following item to the 'Fundamental Responsibilities' section: "Consider the well-being of society in matters affecting health" [20]. This addition, however, does little to address, in any substantively meaningful way, the duty to care obligations of HCPs in the context of an infectious disease outbreak. Does the addition of this responsibility obligate physicians to provide treatment even when doing so would put their own health in peril? The wording is too vague to be of any significant guidance in clinical practice.

In contrast, the American Medical Association (AMA) appears to have recognized the present need to address the issue of duty to care. In the wake of the 9/11 terrorist attack, the AMA has adopted several new ethics policies that focus specifically on the medical profession's obligations and responsibilities in the context of a public health emergency. The following passage is from the AMA policy document "Physician Obligation in Disaster Preparedness and Response" that was adopted in June 2004:

"National, regional, and local responses to epidemics, terrorist attacks, and other disasters require extensive involvement of physicians. Because of their commitment to care for the sick and injured, individual physicians have an obligation to provide urgent medical care during disasters. This ethical obligation holds even in the face of greater than usual risks to their own safety, health or life. The physician workforce, however, is not an unlimited resource; therefore, when participating in disaster responses, physicians should balance immediate benefits to individual patients with ability to care for patients in the future." [21]

While the AMA has taken a step in the right direction by stating the obligations of its members, and it is to its great credit for initiating this process, it remains to be seen whether other national medical associations and other health care professions will follow suit and redress the silence of codes of ethics on the duty to provide care.

### Have codes of ethics always been silent on the duty to care?

To some extent, codes of ethics can be seen as reflections of enduring professional values. At the same time, they are also clearly influenced by and are the product of historical circumstances. The CMA, for instance, previously included in its *Code of Ethics *a strongly worded statement explicitly addressing the obligations of physicians in infectious disease outbreaks. The 1922 version reads as follows: "When pestilence prevails, it is their [physicians'] duty to face the danger, and to continue their labours for the alleviation of suffering, even at the jeopardy of their own lives" [22]. This is the only appearance in the CMA *Code *of this type of strong categorical language regarding the professional duty to care. Interestingly, the specific text cited above appears for the first time in the revision following the 1919 influenza pandemic and then, conspicuously, disappears from the next revision released in 1926.

The AMA included the very same provision in its *Code of Ethics *from 1846 through until the 1970s when it was likewise excised. This marked professional retrenchment from a strong obligation to provide care – as reflected in current codes of ethics – is attracting increased interest of late. A number of explanations have been proposed by academic commentators. For instance, the retrenchment has been linked to the rise of government and corporate intrusions into medical practice [23]. Others, including Clark [12], have pointed to an increasing general belief originating circa 1950 that infectious diseases had been vanquished. It is most likely the case that both these factors played a significant role in the observed retrenchment over time.

Irrespective of the reasons underlying the current silence, there can be little doubt that infectious diseases are an increasing clinical reality in the developed world, and have long been a tremendous challenge in the developing world. For this reason alone, the continuing silence of codes of ethics is greatly problematic, both clinically and normatively.

### How strongly formulated should the duty to care be?

There is no current consensus as to how explicitly and stringently the requirements for the duty to care should be stated [14]. In a 2003 survey of 1000 American physicians, respondents reported decidely mixed views on whether they would continue to care for patients in the event of an outbreak. Given that only a narrow majority of the surveyed physicians reported believing in a professional duty to treat patients in epidemics, the authors of the study concluded that there should be a reinforcement of "the [medical] profession's ethical duty to treat" in the event of a public health crisis [10].

This call for the reinforcement of the duty to care echoes the 1922 *CMA Code of Ethics *that clearly stipulates that physicians have a duty to provide care, even at the jeopardy of their own lives. This statement may indeed be considered too strong and too categorical by many today. To require the provision of care even when doing so entails significant risk to the provider would appear to be demanding that all HCPs behave like "supreme Samaritans" [12]. Is this reasonable? Is this ethical? These remain open questions, but it is doubtful that all HCPs would adhere to such stringent obligations when faced with a SARS-like crisis. As Emmanuel [24] has instructively noted, the historical record of physicians is decidedly mixed in this regard; indeed, it was in response to the vocal and mounting opposition to treating seropositive patients at the height of the HIV/AIDS epidemic in the 1980s that the medical profession reconsidered the on-going retrenchment of the duty to care. With the threat of a new epidemic, another round of full and open discourse is required as to whether the acceptable standard of professional engagement should occur at the level of "supreme", "good", or "merely decent" Samaritan [12].

### What next?

There is presently a need to address the professional duties of HCPs to their patients with the risks to the well-being of society, which may include family, friends, co-workers, and other patients, in addition to the population at large. This is a matter of balance. The content of current professional codes of ethics offers little guidance or reflection of consensus in the health care community. In the wake of SARS and with the current threat of avian influenza, it is clearly time for medicine, nursing, and other self-regulating health care professions to address the issue head on.

A number of options are open to the professions. One option, which we have already argued is unacceptable, would be to remain silent. On the other extreme, codes of ethics could be revised with a strong emphasis on the professional obligation and duty to provide care during infectious disease emergencies; that is, assume a position leaning towards supererogation, or performing acts that are 'above the call' of duty [7]. Several other options exist between these two extremes. For instance, the codes could reflect a strong but limited duty to care, with the limits clearly specified. Alternatively, there could be a weak emphasis on duty to care – an option more sympathetic to the self-regarding concerns of HCPs [8] – although this may run the risk of dissolution of the generally high regard for the health care professions that exists in society today.

We maintain that, with respect to the duty to care, it is not acceptable for codes of ethics to be vague, ambiguous, or otherwise avoid explicit statements of position. This is particularly true in light of calls for additional protections for HCPs during infectious disease outbreaks, including a position statement to that effect issued by the Ontario Medical Association [25]. Such calls for danger pay and/or enhanced disability insurance could be justified if the professions expressed a strong commitment to the ethical duty to provide care during public health emergencies. In the absence of such commitment, however, any additional measures to protect and safeguard the well-being of HCPs would appear self-serving and misplaced.

In the current context of pandemic influenza planning, as with other public health emergencies, there is an acute need for strategies to encourage greater discussion and dialogue among all interested parties and stakeholders [26]. A first step would be for the professional colleges to create a forum to engage their memberships and encourage the exchange of views on the issue. Such an exchange could then inform the development of formal position statements on the duty to care during communicable disease outbreaks, as well as the development of clear and unambiguous guidelines regarding the professional rights and responsibilities and the ethical duties and obligations of HCPs during such outbreaks. Such statements ought to be made publicly available (e.g., prominently posted on the websites of professional health care colleges and associations) in order to encourage sustained dialogue on the issues raised by professional colleges.

A next step would be to foster public debate and dialogue on the positions taken by the various health care professions. To promote this public debate, a working group at the University of Toronto Joint Centre for Bioethics has produced an ethical framework to guide preparedness planning for pandemic influenza, based in part on experiences and study of the SARS crisis [27]. The framework presents a 15-point, value-based ethical guide for pandemic planning, including the value of duty to care. This report has been made publicly available via the internet and the use of webcasting and electronic town hall meetings are being planned to facilitate an open exchange. It is of utmost importance to promote a public discourse on these issues and, most importantly, to give a voice to all those who would be directly affected by a communicable disease outbreak. Together, health care professionals and the general public should participate in discussions to determine whether and when it is legitimate for HCPs to eschew the duty to care in the face of personal risk.

## Summary

In light of the recent experience of Canadian physicians, nurses, and other HCWs on the frontlines of the SARS outbreak, we submit that the Canadian health care community should lead the charge to address issues of duty to care and ethical obligations in times of public health emergencies. In place of open and honest discussion, we currently have vagueness and ambiguity. In our view, health care codes of ethics should speak specifically to this issue in order to guide professional behaviour during infectious disease outbreaks. Indeed, the time to address the ethical duty to provide care is at hand – before the arrival of the next public health emergency.

## Abbreviations

HCPs (health care professionals); HCWs (health care workers); CMA (Canadian Medical Association); AMA (American Medical Assocation).

## Competing interests

The author(s) declare that they have no competing interests.

## Authors' contributions

CR drafted the first version manuscript. CST contributed to the drafting and subsequent revisions of the manuscript. CMB, MB, LH, and RZS contributed to subsequent revisions of the manuscript. REGU conceived the original idea for the paper, participated in every stage of its development, and will act as guarantor. All authors have read and approved the final version of the manuscript.

## Pre-publication history

The pre-publication history for this paper can be accessed here:


